# New Procedure for the Kinematic and Power Analysis of Cyclists in Indoor Training

**DOI:** 10.3390/s20216135

**Published:** 2020-10-28

**Authors:** José Antonio Calvo, Carolina Álvarez-Caldas, José Luis San Román, Ramón Gutiérrez-Moizant

**Affiliations:** Mechanical Engineering Department, Institute for Automotive Vehicle Safety (ISVA), Universidad Carlos III de Madrid, Leganés, 28911 Madrid, Spain; calvarez@ing.uc3m.es (C.Á.-C.); jlsanro@ing.uc3m.es (J.L.S.R.); ragutier@ing.uc3m.es (R.G.-M.)

**Keywords:** chainrings geometry, cyclist biomechanics, error ellipses, power meter, cyclist performance

## Abstract

In this research, the performance and movements of amateur and professional cyclists were analyzed. For this, reflective markers have been used on different parts of the body of the participants in conjunction with sports cameras and a mobile power meter. The trajectories of the markers have been obtained with the software Kinovea and subsequently analyzed using error ellipses. It is demonstrated that the error ellipses help determine movement patterns in the knees, back, and hip. The covariance of the error ellipses can be indicative of the alignment and symmetry of the frontal movement of the knees. In addition, it allows verifying the alignment of the spine and the symmetry of the hip. Finally, it is shown that it is necessary to consider the uncertainty of the power devices since it considerably affects the evaluation of the cyclists’ performance. Devices with high uncertainty will demand a greater effort from the cyclist to meet the power required in the endurance test developed. The statistical magnitudes considered help to analyze power and evaluate the cyclists’ performance.

## 1. Introduction

Cycling is in continuous technological development, due to the high competition of this sector and the greater demand of the customer, who is always looking for the lightest and most advanced product.

On the other hand, while the technical innovation of the bicycle grows, the interest in the study of the biomechanics of cyclist is also rising [1]. This knowledge allows to maximize the cyclists’ performance and avoids possible injuries [2,3,4]. Several studies have been developed on the position of the foot on the pedal, the height of the saddle, the displacement of the saddle and its inclination, the correct position of the steam and the handlebar, etc. [5,6,7].

The efficiency of pedaling and the minimization of knee injuries due to unwanted movements during the loading phase have been extensively studied in recent decades [8,9,10]. Because of these biomechanical studies, a chainring with a non-circular geometry was developed.

The oval chainring was born as a biomedical need to reduce the stress peaks suffered by the knee when pedaling. Bisi et al. [11] showed that oval chainrings allow for reductions of 7% in the knee flexion joint moments with respect to the circular chainring without important difference in metabolic and kinematic data. Ranking et al. [12] developed a theoretical analysis in order to find the optimal chainring geometry. The optimal non-circular chainring found by the authors [12] could increase the crank power output, since it allows the muscles to transmit a greater effort for a longer time during the power stage of the cycling cycle. Additionally, they concluded that the oval chainring geometry might reduce the knee injuries. However, Sinclair et al. [13] advise that the geometric chainring configurations do not appear to influence the patellofemoral force per 20 km joint during the pedal cycle. They found that the difference between the knee forces are not significant (around 8%) comparing the circular chainring with the oval one.

Another reported benefit of using the oval chainring is a greater involvement of the large muscle groups in the lower body in power development [14]. Duc et al. [14] showed that the muscular activation of the mono-articular hip and knee extensor muscles is slightly increased (~5–15%) when the cyclists pedaled at 70 rpm using the oval chainring. The activation of large muscle group did not significantly influence the physiological response (heart rate and oxygen consumption) as compared to a traditional chainring [15].

During pedaling, it is desirable for the athlete to try to optimize the recruitment of large muscle groups to produce pedal power [16]. Professional cyclists have the ability to distribute the effort in the pedaling cycle in high cadences compared to the amateur ones [17,18]. This is probably due to the fact that professional cyclists have the greater facility in employing some muscles and alleviating the effort of others, performing a more effective muscular recruitment with less variability [18]. However, the muscle recruitment patterns depend on the muscle fatigue, cadence change, and cyclist posture [19].

According to the elite amateur cyclist test’s impressions, when the non-circular chainrings are used, the power delivery seems to be more continuous than with circular ones [20]. Therefore, it could be hypothesized that the continuous pedal action associated with the oval chainring would generate energy savings [20]. If this can be demonstrated, the use of the oval configuration could be beneficial, especially in situations where maximum effort is required [20]. However, the muscle coordination together with regulation settings of oval chainring could be key in the improvement of the rider performance [14,20,21].

Cyclists that use the conventional chainring need a kinematic adaptation period (generally a few minutes) to become familiarized with the use of the oval chainring [22,23,24]. However, there are differences between the perceptions of the effort made with this type of configuration, especially in younger athletes or amateurs [15,21,25]. Therefore, the subjects’ cycling experience could be another important variable in the effects of the oval chainring on the performance. According to Lucia et al. [26], well-trained cyclists do not take advantage of the oval chainring as they are too familiarized with traditional geometry.

Usually, the biomechanical studies need instrumentation that is not only complex, but also, in some cases, uncomfortable to the athlete (instrumentation with cables that need to be attached to the body of the cyclist). Recently, wireless technology (inertial measurement units and microelectromechanical sensors) has been used in different sports disciplines, such as American football, swimming, and others [27,28]. The principal advantage of these measurement instruments is the possibility to monitor athletes in a real sport environment. Additionally, the measurement devices are small, lightweight, and unobtrusive, which allows full movements of the athlete in each sport discipline [27,28].

Optical measurement systems, such as motion capture cameras, allows obtaining full-body capture, but are only suitable under controlled laboratory conditions, since multiple images of the athlete’s movement without any type of obstruction are needed [28]. Bernardina et al. [22] suggested the use of the sport video cameras to analyze biomechanical human movements. Despite the fact that the accuracy and precision of these motion capture devices are significantly different from the specialized ones, the authors [22] demonstrated that such differences have minor practical effect on reconstructed three-dimensional (3D) kinematics.

According to the state-of-the-art, much research has been done on the performance of cyclists, but little attention has been paid in the determination of the movement patterns of the body and muscle coordination. Investigation into kinematics could identify such biomechanical variables that affect the cyclist performance [29]. In the actual research, the patterns movement of two groups of cyclists: professionals and amateurs, were studied. For this, sport video cameras and potentiometer crankset have been used. The methodology used in the analysis of the cyclists’ body trajectories allows a better visualization of their posture and helps to identify possible unwanted movements. It is shown that the error ellipses obtained from the cyclist movements plays an important role in the verification of the alignment of the spine, the alignment and symmetry of the hip, and helps to better visualize the frontal movements of the knee.

In recent years, the use of portable power meters to evaluate the performance of cyclists has been increased. However, there is no definitive procedure that indicates how to analyze the power data from these devices [30]. In this study, a new procedure for the analysis of power data is proposed, which helps to evaluate the performance of the cyclist (especially in indoor training sessions or when the change of the boundary conditions during training are not important). It is recommended to consider the inherent uncertainty of the power measurement equipment, because it affects the performance results of the test. Finally, some recommendations are given to evaluate the cyclists’ performance based on the quantification and interpretation of some statistical variables.

## 2. Materials and Methods

The study was carried out on two groups of male cyclists. The first group consisted of two amateur cyclists, who perform physical activity on the bicycle more than 3 times per week. The age of the amateur cyclists is 25 years old, they have a weight of 75 ± 1 kg and an average height of 1.75 ± 0.01 m. The second group consisted of two professional cyclists with more than 20 years of experience, who have participated in national and international competitions. The age of the professional cyclists is 40 years, their average weight is 68 ± 0.5 kg and a height of 1.74 ± 0.02 m. The amateurs have a body mass index of 24 ± 0.3 kg/m^2^ and the professionals of 22.5 ± 0.2 kg/m^2^.

It is important to advise that the aim of the actual research work is to use a new methodology that facilitates the study of the movement patterns of cyclists, as well as the analysis of the power developed by them. Therefore, the movement patterns as well as the power developed cannot be generalized to other individuals, since it is necessary to increase the sample. However, when evaluating different groups as well as using different configurations of bicycle chainrings, it has allowed us to verify if the proposed methodology is capable of detecting the changes introduced by the variation of the boundary conditions.

### 2.1. Equipment

#### 2.1.1. Capture Motion Devices

To make the recordings of the movement of the cyclists, it was decided to use sports video cameras instead of commercial motion cameras for the following principal reasons: Their lowest cost, this point is important as several simultaneous recordings are needed. In addition, this type of study is intended to be reproducible at any level of cycling experience, for example, to verify and improve the technique used by amateur cyclists without needing to go to specialized or high-performance centers. Another reason is the possibility of recording for long times and with an adequate recording speed (between 60 to 120 frames per second), which is similar to commercial motion capture cameras [31]. According to Bernardina et al. [22], the accuracy of these cameras is adequate for the applications in biomechanics.

In the actual research work, three different cameras have been used. A common sampling rate of 60 frames per second and video resolution of 1080p has been set. All cameras were verified to have lens distortion correction enabled (normal lens setting). In this way, the cameras perform a software-level correction for fisheye distortion. In addition, we checked that the areas of interest were far from the edges of the captured images because the lens distortion correction loses quality at the edges. For the location of the cameras, the recommendations of the different authors consulted were considered [32,33,34]. The cameras were placed in a radius measured from the center of the bicycle of 3.5 m and at a height coinciding with the midpoint of the body of each participant.

The sport cameras used were:

- For the front record, a GoPro Hero 5 Black camera was used. This is a high-quality sport camera with an image quality of 4 K at 30 frame per second or 1080p at 120 frame per second.
- For the side view, a GoPro Hero 5 Session camera was used. This camera is considerably smaller than the previous one, and is capable of recording in 4K image quality. It has several recording speeds from 24 to 100 frames per second.
- For the back view, we used a low-cost Xiaomi Yi sport camera with a maximum recording speed of 60 frames per second with a 1080p quality.

#### 2.1.2. Power Meter Device

**Figure 1:**
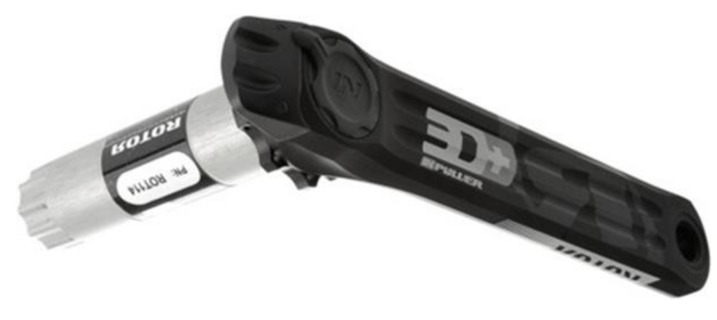
Rotor INPower Crank.

To analyze the pedaling performance of the cyclists, INPower Rotor Cranks power meter (Figure 1) has been used. The power meter is integrated in the crank axle; therefore, it is protected from the environmental condition and unexpected impacts. The device uses a set of strain gauges connected to a complete Wheatstone bridge that allows measuring the applied torque during the pedaling motion without temperature changes affecting the measurement. A small accelerometer included in the power meter detects the movement and the position of the crank to determine the angular speed. With this information, the instantaneous power generated is obtained. According to Abbiss et al. [35], the validity of the power meter can be high if the power output is calculated as mentioned before. The signals are transmitted by a small radio frequency according to the ultra-low power wireless protocol [36]. The sampling frequency of the force is 200 Hz and the power of a rotation is emitted 4 times per second.

Maier et al. [37] have carried out an exhaustive accuracy verification of 54 mobile cycling power meters from 9 manufacturers (including Rotor). The authors concluded that the current power meters vary considerably in their trueness and the precision is generally high but differs between manufacturers. The mean deviation and the coefficients of variation obtained from the Rotor power meter were 2.1% ± 0% and 0.4% ± 0%, respectively [37]. Considering previous authors’ advices, in the actual research work, we assumed a conservative accuracy for the power meter device of 2.5%.

The INpower was calibrated for each participant, in order to compensate for any mechanical change that could influence the correct power measurement. The calibration was made following the instructions of the user manual, after assembling the bike with all its accessories, including pedals and without any additional weight on them. It is important to advise that with this power meter, only the left foot action be measured, because a perfect balance is assumed of the cyclist pedaling with 50% for each leg.

All cyclists used the same bicycle and the contour conditions were common for all (indoor condition). The bicycle used in the tests was a Giant TCR. To recreate changes on the road such as slopes, the bicycle was fixed into a BKOOL roller [38]. This equipment generates resistance in the rear wheel and allows simulating real cycling conditions in indoor training.

A Garmin heart rate sensor was fixed on the cyclists’ bodies to evaluate the heart rate during the test. INPower software has been used for the acquisition and analysis of the power data from the cranks and heart rate sensor.

#### 2.1.3. Movement Analysis Software

For the analysis of the movements of the cyclist, different markers were placed on the body zones of interest. For this, an elastic adhesive white band model kinesiology of 3 cm in diameter has been selected. The size of the markers is within the range used in previous studies [29,34,39]. To ensure the reliability of the measurements, all participants were asked to wear contrasting clothing, i.e., black clothing.

The trajectories of several markers placed on the cyclists’ bodies were obtained by Kinovea software. This is a free open software used for analyzing the technique of the different sport skills. According to Buscà [40], Kinovea is the most commonly used software in the sports field, and incorporates a set of tools to study and compare measured technical performances. This software has been used in sports studies and in clinical fields, as well as a tool to test the reliability of other novel technologies [32,33,34]. According to the statistical analysis carried out by the authors [32], the Kinovea software is a precise and reliable program with which to obtain angles and distance in the perspective visual range from 90° to 45° and at a 5 m distance from the recorded object. However, for optimum results, they suggested a capture angle of 90°. In the research works developed in the sports field [33,34], it was demonstrated that the measurements developed by the Kinovea software are in agreement to the those made with sophisticated measurement equipment.

**Figure 2:**
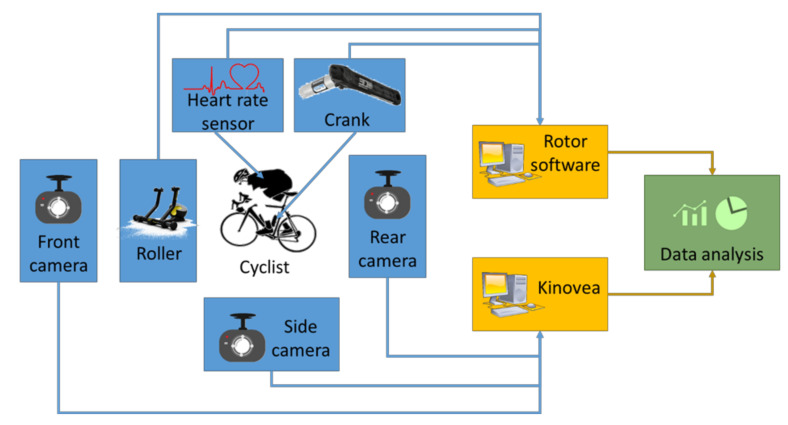
Schematic representation of the experimental set-up of the indoor test.

According to the conclusions of the different authors consulted [32,33,34], in this research work, it has been considered that the Kinovea software is suitable for the biomechanical study that has been carried out. The schematic representation of the experimental set-up developed in the actual research work is shown in Figure 2.

### 2.2. Measurement Points

**Figure 3:**
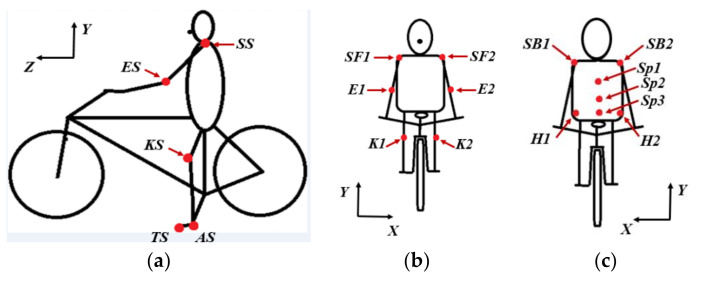
Measurement points. (**a**) Side view, (**b**) Front view, (**c**) Rear view.

Figure 3 shows the tracking zones analyzed in the actual research work.

#### 2.2.1. Side View

The main points analyzed in the side view (Figure 3a) were:

- Knee: According to the different sources reviewed [2,9,41], the knee is the joint that supports the most workload during pedaling. Consequently, knee injuries in cyclists are very common due to their overload during the pedaling movement [2,3]. This is why, in the present research study, the influence of the use of different chainring type geometry in the correct knee movement will be analyzed. The objective is to verify if the oval chainring can prevent dangerous or anomalous movements and, therefore, reduce the risk of pain during cycling and future injuries.
- Ankle and toe: these two points will provide information on possible energy losses due to a turn of the foot around the ankle. This turn should not occur. Both exaggeration of the ankle movement (dorsiflexion or the plantarflexion) of the foot when pedaling can increases the risk of Achilles tendon pathology [2].
- Shoulder: it is important to check that the shoulders do not move excessively up and down during pedaling. This situation usually occurs when the cyclist is very tired [2]. It is also essential to check the flexion of the lumbar spine to avoid injury [2]. According to the investigation carried out by Clarsen et al. [42], the lower back pain is the most common medical attention injuries in professional cyclist with a 45% of all medical attention injuries. The results was constructed by the interview of 116 elite road cyclist.
- Elbows: this measurement point will be used to control the vertical movement of the elbows. The vertical movement will help to verify whether flexion of the upper body occurs, increasing the risk of low back pain.

#### 2.2.2. Front View

Measurement points in front view (Figure 3b) were:

- Knee: From this point of view, it is necessary that the hip, knee, and ankle should be aligned to reduce the risk of knee pain, because an excessive deviation from the vertical line contributes to a range of knee injuries [43].
- Shoulders and elbows: Their displacements in the vertical axis can indicate an exaggerated swing movement of the cyclist’s trunk, which can increase the risk of injuries in the lower back [2].

#### 2.2.3. Rear View

The tracking zones from the rear view (Figure 3c) were:

- Spine: Large vertical movements could indicate multiple flexion of the back and increase the risk of lumbar overuse injury [2]. On the other hand, asymmetrical riding position can inform us that the cyclist probably has leg length inequalities or other anatomical anomalies, such as a scoliosis [44].
- Shoulders: It is important not to register pendulum movement, since it implies a loss of energy and increases the risk of lower back pain [2].
- Hip: The recurrent changes in movement of the hip on the saddle directly affects lumbar posture and can indicate the fatigue of the larger muscles, such as hamstrings, quads, and calf muscles [45]. Other possible causes may be that: the saddle height is not correct, the handlebar is too low, the crank length is too long, or simply due to poor coordination and posture control [45]. The greater alignment of the lower extremities increases the fatigue resistance of the cyclist and increases the output power [46].

### 2.3. Test Routine

**Figure 4:**
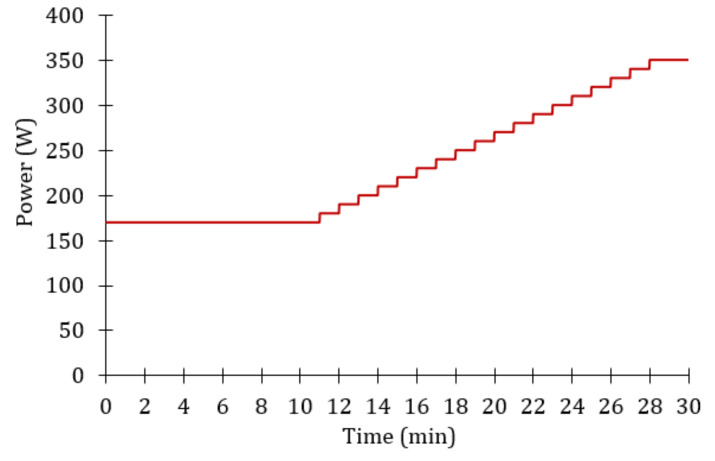
Schematic representation of the power test routine.

The routine test developed is shown in Figure 4. This routine consists of three effort stages and is similar to that used in the endurance test of professional cyclists. Before starting the test, the participants were instructed to assume a natural position, similar to the one they usually adopt when pedaling, but without being able to separate from the saddle. The stages of the test are described below:

- Effortless stage: Warm-up stage with a pedaling power of 170 ± 10 W for 10 minutes. This stage was intended to avoid possible back or abdominal injuries.
- Progressive effort stage: Intermediate zone of the event. At this stage, cyclists were asked to increase their pedaling power from 170 to 350 ± 10 W, at a speed of 10 W per minute.
- Maximum effort stage: The last stage of the test, with a duration of 2 minutes. The required power reaches its highest value (greater than 340 W). In this phase, resistance to fatigue depends on the physical condition of each of the participants, but it can also depend on the pedaling technique used.

The endurance test was performed once per participant. During the test, each cyclist was asked to try to comply with the required power as far as possible, since the boundary conditions were far from their usual conditions.

## 3. Results and Discussion

The majority of the movements analyzed correspond to the mean values of both cyclist groups. However, detailed analyses were performed for those cyclists who showed unusual movement patterns compared to the other participants. For the analysis, the recommendation for standardization in the reporting of kinematic data was followed [47]. According to this report, the majority of the biomechanical studies use the Cartesian approach as a coordinate system. Additionally, it is indicated that it will be left to the investigator to devise systems for the reporting of more specialist situations. For this reason, we proposed the representation of some movement patterns through error ellipses, because we believe that it allows a better comparison of results and helps to identify unwanted movements.

### 3.1. Knee Movement

The movement of the knees has been studied with the recordings of front and side view. For this, the standard deviation of all the images captured was considered.

To verifying the alignment of the knees with the vertical axis, the uncertainties of the vertical and horizontal movements have been represented through error ellipses, for a confidence level of 95% [48]. In this way, if there is no covariance between both movements, that is, if they are independent, the axes of the ellipse will coincide with the vertical and horizontal axes. Therefore, it can be concluded that the knee movement is aligned with the vertical axis. In the opposite case, if the covariance between both movements is confirmed, the axes of the error ellipse will be inclined, which could indicate that the knee is not aligned with the vertical line. From this point of view, it is essential that the trajectory of the knee be as close as possible to a vertical movement to prevent future knee injuries [43]. Therefore, the major axis of the error ellipse calculated has to be aligned with the vertical direction.

**Figure 5:**
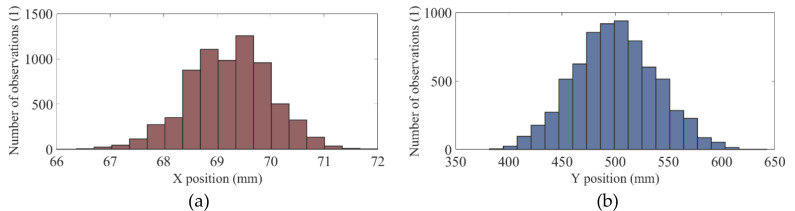
Histogram of (**a**) position X and (**b**) position Y of the professional cyclist 1, left knee marker.

To apply this method, it is important to verify that the data resembles a normal distribution. We believe that it is not necessary to guarantee that the data distribution is normal; therefore, visual inspection of the distribution may be adequate. Figure 5 shows as an example the probability histograms obtained with the displacements of the professional cyclist 1, left knee marker *K2*.

**Figure 6:**
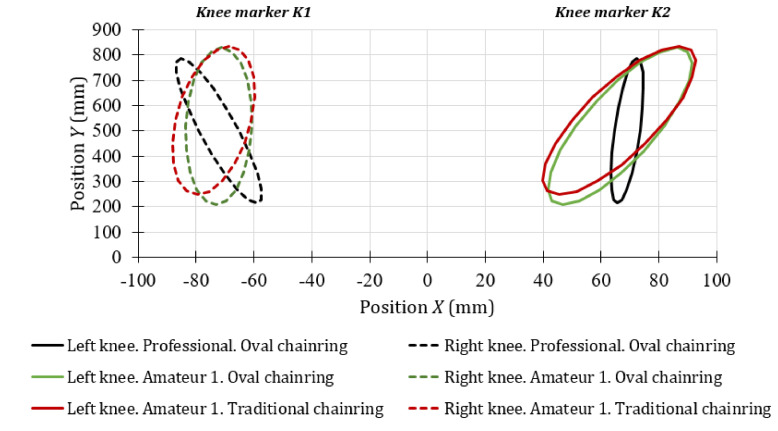
Front view of the knee markers’ movement during the minimum effort for the cyclist 1 and the professional cyclists.

Figure 6 shows the error ellipse of the trajectory of the knee for the first stage of the test, for the amateur cyclist 1 and the professional cyclists. These results were calculated from the successive image captures made by the front camera. The right knee is represented on the left side of Figure 6 and the left knee on the right side. The error ellipses shown define the region that contains 95% of all possible knee positions observed during the test. The error ellipses have been drawn with the length of the major axis and the length of the minor axis of $2\sqrt{c\cdot \lambda_{1}}$ and $2\sqrt{c\cdot \lambda_{2}}$ respectively, where *λ_1_* and *λ_2_* are the eigenvalues of the covariance matrix and *c* is the scale factor for the chi-square distribution [49]. The shape of the error ellipse does not depend on the scale factor *c*, since it is defined by the variances of the movements of the markers. However, it is recommended to use a scale factor corresponding to a 95% confidence level to better visualize the ellipses as they increase in size.

The results shown in Figure 6 allow visualizing the contours of the error ellipse instead of a cloud of points of the positions of the knee markers *K1* and *K2* (see Figure 3b). As can be seen, under the minimum effort stage, the possible movements of the knees of both amateur number 1 and the professional cyclists are not symmetrical with respect to the vertical axis. In addition, it can be noted that there is a covariance between vertical and horizontal movements, so it can be assumed that there is a slight inclination of the knees with respect to the vertical axis as a function of the magnitude of the covariance. The movements of the amateur cyclist 1 are very similar in conditions of minimum effort for both chainring geometries. However, a variation in the inclination of the knee for this cyclist has been observed depending on the type of chainring used. For the amateur cyclist 1 with circular chainring, the angle of the major axis of the error ellipse with respect to the vertical line was 4.03° for the left knee and 1.08° for the right knee. On the other hand, when the amateur cyclist 1 used the oval chainring, the angles were 3.63° and 0.17°, respectively.

According to the angle of the semi-major axis of the error ellipses shown in Figure 6, the possible knee movements for the amateur cyclist 1 were an external rotation of the left knee and internal rotation of the right one. This indicates that this subject was probably not positioned correctly in the saddle during this stage of the test. On the other hand, it was observed that the horizontal movements of the professional’s knees are the lowest of all, because the major axis of the error ellipse is much larger than the other axis. Therefore, as was expected, the path of the professional cyclists was nearest to the vertical line. In this case, the angle of the vertical trajectory was –2.67° for the right knee and 0.67° for the left one. According the inclination of the error ellipses, the professional cyclist tends to make a slight external rotation of both knees.

**Figure 7:**
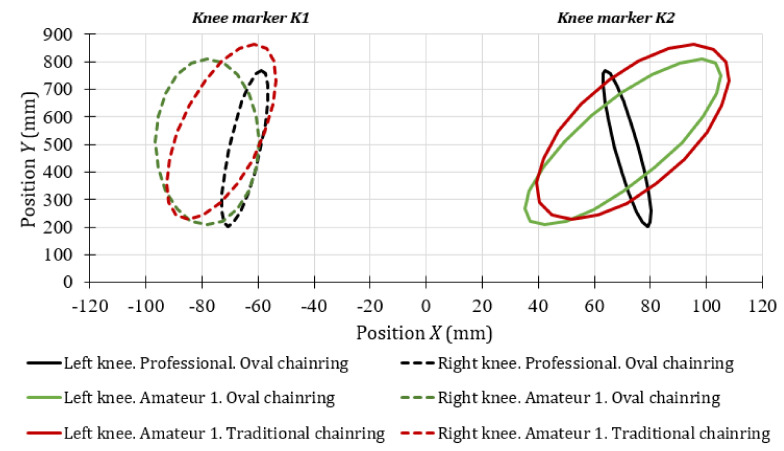
Front view of the knee movements during the maximum effort for the amateur cyclist 1 and the professional cyclists.

Figure 7 shows the error ellipses, for a confidence level of 95%, corresponding to the last stage of the power routine test performed. In this case, it was also visually verified that the histogram of the knee movements resembles a normal distribution. From the results obtained in this last stage of the test, it could be confirmed that the posture of amateur cyclist 1 was similar during the entire power test. However, an important improvement was observed in the frontal movement of the right knee when the amateur cyclist 1 used the oval chairing (K1 knee marker, green dotted line). In this case, the alignment of the knee with the vertical axis was perfect. The angle of the error ellipse with the vertical axis reduced from 5° with the circular chainring to 0.03° with the other one.

The analysis of the frontal movement of the left knee of the amateur cyclist 1 (*K2* knee marker, solid green line from Figure 7), indicates that the external rotation is larger when the oval chainring is used. The offset angle with respect to the vertical line increases by approximately 45%. The excessive medial motion of the knee in the frontal plane may also be a risk factor for future patellofemoral pain [43].

Regarding the frontal trajectories of both knees of the professional cyclists, it can be seen in Figure 7 that there is an almost perfect symmetry of the error ellipses with respect to the vertical axis. The angles of the error ellipses in this case are –1.52° for the left knee and 1.22° for the right knee. Therefore, it can be assumed that in the maximum effort condition, professional cyclists tend to improve their posture.

Regarding the sagittal trajectory of the knees, ankles and toe of the participants, no significant changes were observed and that is why the results are not presented. Furthermore, the proposed analysis methodology has no practical application in the study of the sagittal movement of the mentioned body zones.

### 3.2. Shoulders and Elbows Movements

**Figure 8:**
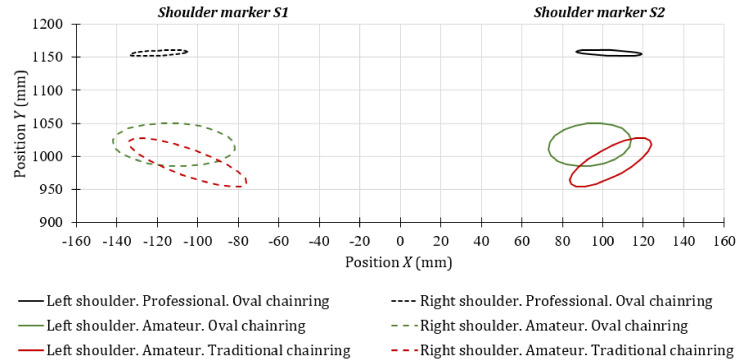
Front movements of the shoulder markers for the maximum effort condition.

**Figure 9:**
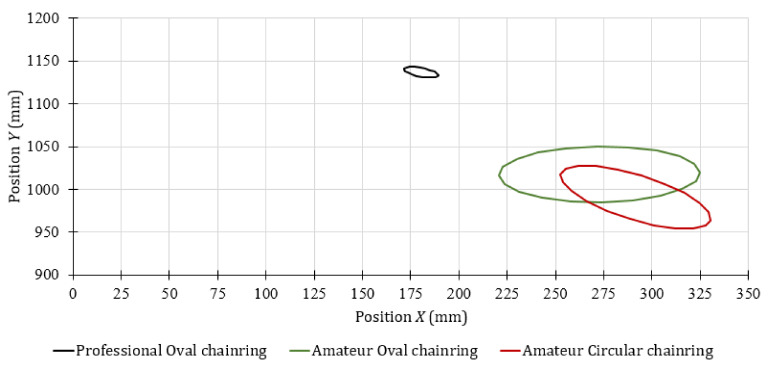
Shoulder marker *SS* side movement for the maximum effort condition.

Figure 8 and Figure 9 show the front and side movements of the cyclists’ shoulders, which have been represented through error ellipses. For its application, it was necessary to verify that the probability distributions of the movements in the X and Y directions were similar to the normal distribution. The axes of the error ellipses used in the study of the marker shoulder help determine the position adopted by the cyclists during the test. The major axis of the ellipse indicates the greatest variability of the displacements, while the minor axis is the one with the least movement variation. If there is covariance between the two motions, the resulting error ellipse will be inclined.

According to the error ellipses corresponding to the front (Figure 8) and lateral (Figure 9) trajectories of the cyclists’ shoulders, the vertical movements’ variation for the case of the professional cyclists is not relevant (less than 25 mm) compared to the amateur cyclists.

In Figure 8, it can be seen that both groups of cyclists tend to swing from one side to the other, but in non-professional cyclists, the movement is more noticeable. On the other hand, according to the tracking made over the lateral shoulder marker (*SS,* from Figure 3a), the amateur cyclists show greater variation in lateral shoulder movements (Figure 9) and adopt a more inclined posture of the back than professional cyclists.

The results shown in Figure 8 and Figure 9 suggest that the oval chainring allowed the studied amateur cyclists to maintain a slightly higher shoulder position. However, it can be seen that the variability of the horizontal and vertical movements with both chainring geometries are similar. Therefore, it can be said that the unwanted movements of the cyclists could depend on their experience. The same trend was observed in the case of elbows.

### 3.3. Back Movement

**Figure 10:**
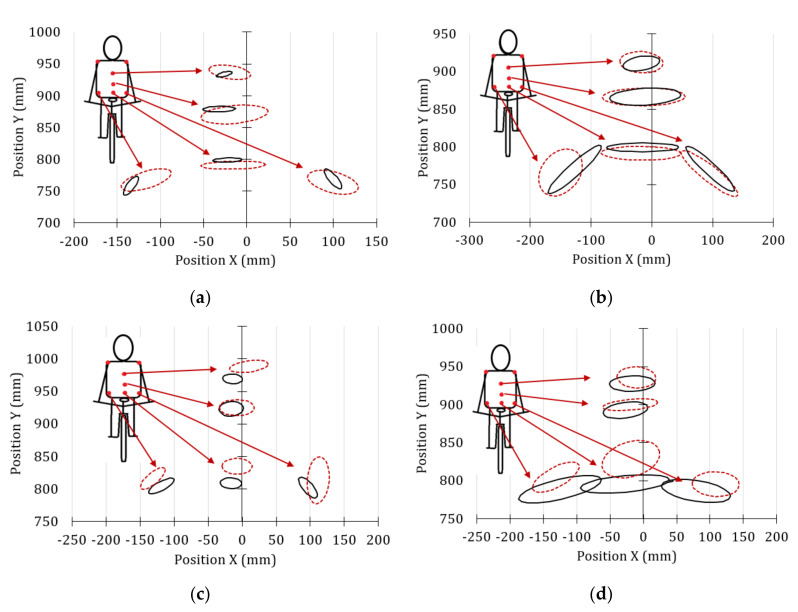
Movements of the back markers of amateur cyclists captured by the rear camera. Traditional chainring = solid line and oval chainring = dashed line. (**a**) Minimum effort amateur cyclist 1, (**b**) Maximum effort amateur cyclist 1, (**c**) Minimum effort amateur cyclist 2, (**d**) Maximum effort amateur cyclist 2.

Figure 10 shows the error ellipse of the lateral and vertical movements of the back markers of the amateur cyclists. The solid line represents the movements when the traditional chaining is used and the dashed line is in the case of the oval chainring. In this case, the error ellipses are used to verify the alignment of the spine and the symmetry of the hip. Ideally, the ellipses of the spine should be aligned and those corresponding to the hip markers should be of equal magnitude, with collinear centers and symmetrical with respect to the vertical axis.

Comparing Figure 10a with Figure 10c, it can be seen that the error ellipses resulting from the back movements of amateur cyclists are smaller when they have used the circular chainring and the effort required is minimal. This means that the movements are more controlled with this chainring geometry. However, with the oval chainring, an improvement in the alignment of the spinal areas has been observed. In addition, the position of the hip points is almost symmetrical for the amateur cyclist 1.

When the effort required is greater (Figure 10b), the variations in movement for the amateur cyclist 1 are quite similar with both cranksets. On the other hand, it has been observed that the variability of movements of the lower back and hips was less in amateur cyclist 2 when he used the oval chainring (Figure 10d). Both amateur cyclists tended to improve their posture regardless of the type of chainring used when the effort required was maximum.

**Figure 11:**
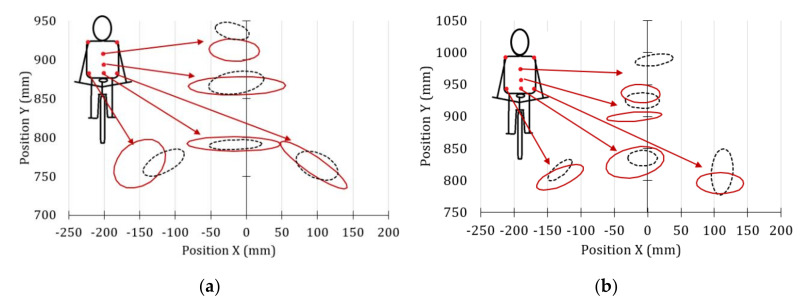
Movements of the back markers of the amateur cyclists when using the oval chainring, for the cases of minimum effort and maximum effort, represented by the dashed line and solid line, respectively. (**a**) Amateur cyclist 1, (**b**) Amateur cyclist 2.

Figure 11 compares the error ellipses obtained from the back movements of both amateur cyclists for maximun (continuous line) and minimun effort (dashed line). The error ellipses inform us that there is a greater dispersion in the movements of the different areas studied when the effort required increases. In addition, it can be seen that the hip ellipses of both cyclists are asymmetric and of different sizes. This could be interpreted as an asymmetry of the hip, especially in amateur cyclist 1, which could lead to an unequal load distribution between both lower extremities. The hip asymmetry of amateur cyclist 1 may be related to the excessive medial motion of the knee in the frontal plane observed in Figure 7. According to the analysis of the movements developed so far, it can be concluded that the amateur cyclist 1 has an unusual posture and a deeper analysis of the hip movements is required to determine if his range of flexion is exceeded [45]. To do this, it would be necessary to relate the error ellipses to the hip flexion ranges, but that is not within the scope of this research. On the other hand, in Figure 11b, it can see that the error ellipses of the amateur cyclist 2 in the maximum effort condition are below those corresponding to the minimum effort. This indicates that the cyclist tends to tilt the torso forward as the effort increases.

**Figure 12:**
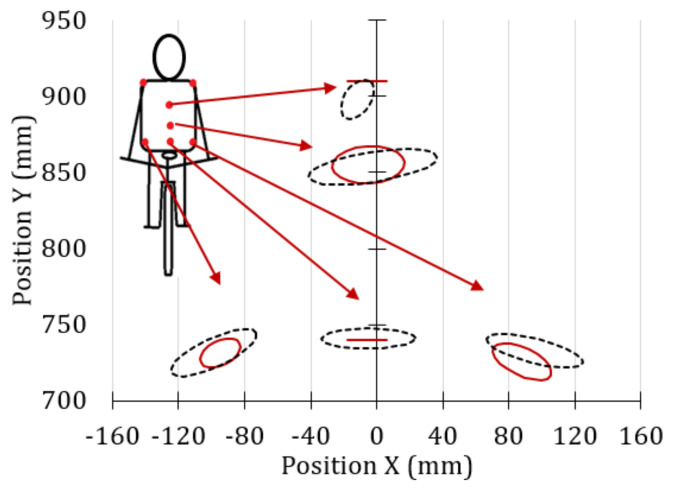
Movements of the back markers of professional cyclists when using the oval chainring, for the cases of minimum effort and maximum effort, represented by the continuous and broken lines, respectively.

Figure 12 shows the error ellipses of the interest zone recorded by the rear camera for the professional cyclists, for the condition of minimum and maximum effort, represented by the solid line and dashed line, respectively.

In Figure 12, it can be observed that in the minimum effort condition, the error ellipses in the upper and lower part of the back do not exist (marker *Sp1* and *Sp3* of Figure 3c), so the ellipses become straight lines. This means that the movements in these markers only occur on the horizontal axis. The vertical position of all error ellipses practically does not change with the level of effort required, so it could be ensured that the upper body stiffness of the professional cyclists analyzed is greater than that of the amateur cyclists (Figure 11). Furthermore, it can be seen that for the maximum effort stage, the error ellipses of the markers of the hip are similar, with collinear centers and symmetric with respect to the vertical axis.

### 3.4. Power Results

Cyclists from recreational to professional generally use power meters to closely check the power output from their training sessions. However, the question is, how can the power data obtained be analyzed? According to Passfield et al. [30], the analysis may consist of a visual inspection to identify the highest power output developed, the number of power intervals completed, or the power output dispersion. Another approach could be to calculate the mean power output for a training session, but also, it is recommended to calculate the standard deviation to know the variability of the power outputs. Mean power with the same value, but different standard deviation, can be indicative of a more stressful race [30].

One approach to study power output data is the exposure variation analysis (EVA) proposed by Mathiassen and Winkel [50]. This method is performed by defining a fixed number of non-overlapping power bins (which represent specific power output intervals) together with a fixed number of non-overlapping time intervals. There is no criterion for setting the time intervals, so different intervals can be defined in the same graph for each power range. The EVA method allows for verifying the amount of time in which the developed power is in the range that has been set.

**Figure 13:**
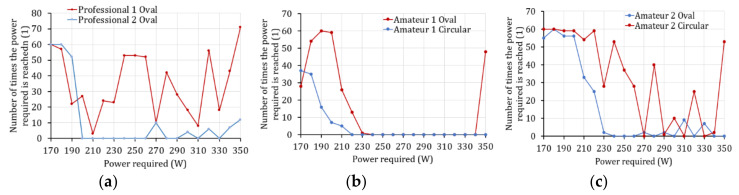
Exposed variation analysis of the powers developed by cyclists. (**a**) Professional cyclist, (**b**) Amateur cyclist 1, (**c**) Amateur cyclist 2.

In the present research work, the power range and the time interval have been defined in the endurance test routine (Section 2.3). Figure 13 shows the EVA of the powers developed by the cyclists studied. The evaluation criterion implemented was to quantify the number of times (frequency) in which the power developed by each participant is greater than or equal to the power required during the test. The power meter recorded one sample per second; therefore, the absolute frequency for each section is 60, except in the maximum effort stage (absolute frequency equal to 120).

The exposure variation analysis shown in Figure 13 allows for verifying the number of times that the participants met the criteria required in the power test. It can be seen that in most cases, the power generated does not reach the value required. Only professional cyclist 1 (Figure 13a) fulfilled the power ranges in at least one instant of time. However, we believe that the results can be better analyzed if they are expressed in terms of relative frequencies.

**Figure 14:**
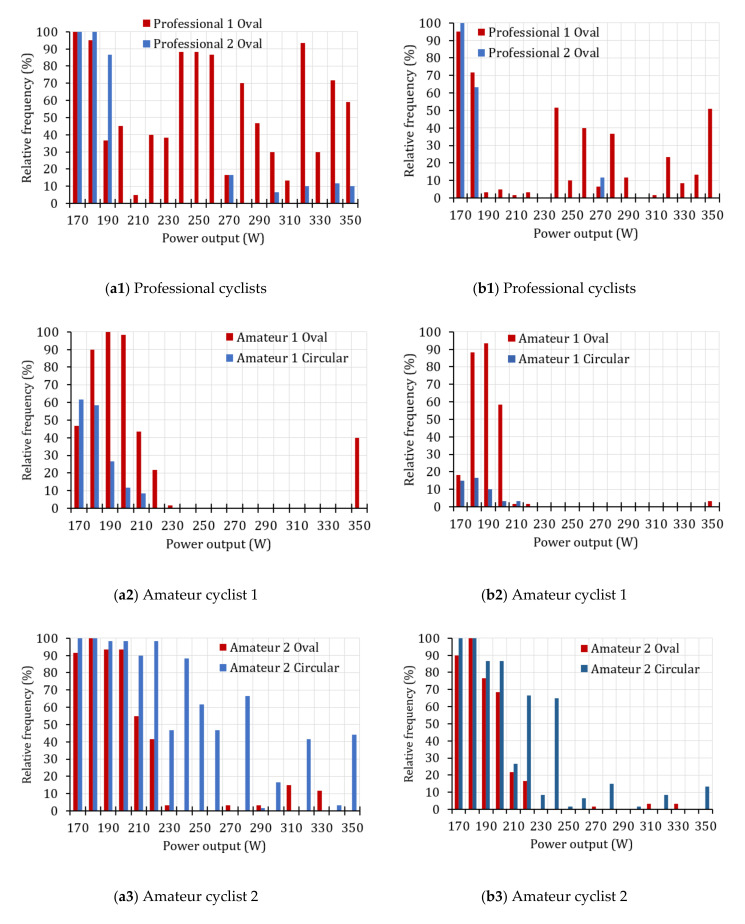
Relative power frequencies developed by cyclists during the effort test. (**a**) Without considering the accuracy of the power meter device and (**b**) considering the accuracy of the power meter.

Figure 14a shows the relative frequencies that reached or exceeded the minimum power required (*P_min_*) in each step of the power test. On the other hand, Figure 14b shows the relative frequencies of the power generated that are greater than or equal to:

(1) $$P_{req}=P_{\min}+\left[k\cdot u\left(P\right)\right]=P-s(P)+\left[k\cdot u\left(P\right)\right]$$

where, *P_req_* is the completed power required that considers the uncertainty of the power meter device, and *P* and *s(P)* are the power value required in each step and its absolute tolerance (10 W, see Section 2.3), respectively. *u(P)* is the uncertainty of the power meter and *k* is the coverage factor for a confidence level of 95% [51].

In Figure 14, relative frequencies equal to 100% indicate that the cyclist fulfilled the minimum required power during the time interval evaluated. On the contrary, relative frequencies of 0% indicate that the cyclist was not able to reach the minimum required power.

According to the results shown in Figure 14, it is important to consider the precision of the power meter, because this significantly affects the distribution of relative frequencies that fulfilled the required power. Devices with high uncertainty will demand a greater effort from the cyclist to meet the completed power required.

The proposed methodology helps to visualize and compare performances between athletes of the same category (Figure 14b1) or analyze performance when using different crankset configurations (Figure 14b2,b3). For example, according to the results shows in Figure 14b2, the amateur cyclist 1 obtained a better performance in terms of power values with the oval chainring than with the circular one. In contrast, amateur cyclist 2 (Figure 14b3) obtained better power results with the circular chainring than with the oval in all stages of the endurance test. Therefore, these non-professional cyclists will have to consider the perception of effort, the fluidity of the movements, and the performance obtained in terms of power generated if they want to select an appropriate chainring configuration.

There is no defined methodology regarding how to interpret power data from training or performance tests when a mobile power meter is used [30]. That is why we are going to analyze in more detail the most relevant statistical parameters, but applied only to professional cyclist 1 because he was the one who showed the best performance. For this, an additional power test has been carried out using the circular chainring.

**Figure 15:**
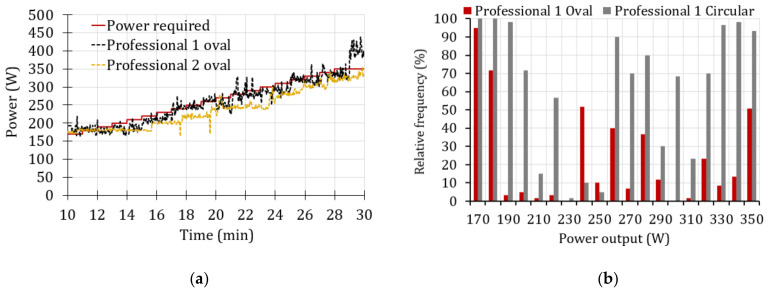
Generated power comparison of the professional cyclist 1. (**a**) Evolution of the power, (**b**) Relative frequencies of the power.

Figure 15a shows the evolution of the powers obtained with the two chainring configurations. On the other hand, Figure 15b shows the distribution of the relative frequencies of the power developed that are greater than or equal to the completed power required (Equation (1)).

Figure 15a shows a similar trend in the power obtained with the two chainring geometries for the professional cyclist 1, so it might be assumed that there is no significant difference between using one chainring or the other. However, the relative frequencies shown in Figure 15b indicate significant differences between the levels of compliance of the cyclist. The level of compliance of the entire test was 24% and 64%, for the oval and circular chainring, respectively. The level of compliance has been determined by dividing the number of developed power greater than or equal to *P_req_* (Equation (1)) by the total number of power data.

Table 1 shows the results of the power analysis in the maximum effort stage of professional cyclist 1, both with the circular and oval chainrings.

In Table 1, it can be seen that despite the fact that the maximum power has been registered when the professional cyclist 1 used the oval chainring, the rest of the statistical parameters (such as the mean, the relative standard deviation, the mode, and the level of compliance) improved when the circular chainring was used. Due to this finding, in the actual research work, it has been considered that maximum power alone is not a good indicator of the cyclist’s performance.

From the results shown in Table 1, it can be deduced that the mean power obtained with the circular chainring is 7% greater than that of the other one. The pedaling quality could be determined by the relative standard deviation that was better when the circular configuration was used (lower dispersion). The statistical mode corresponding to the oval chainring is 20% lower than the circular one, and both have similar relative frequency values. The level of compliance increased significantly (more than 40%) when the circular chainring was used. The score obtained with the circular geometry indicates that only in 7% of the records made did the power developed not satisfy the requirements corresponding to the last stage of the test (Power developed ≥ 359.5 W).

With all these data analyses, it could be concluded that the performance of the professional cyclist 1 was better when the circular chainring was used.

## 4. Conclusions

- According to the results of this research work, the following is concluded: The use of error ellipses is a good tool that provides a better visualization of the frontal movements of the knee and the posture of the upper body. For the knees, the ideal condition is that the error ellipses of the frontal trajectories of both knees are symmetrical and that the major axis is vertical, indicating that there are no abnormal rotations of the knee and therefore that the risk of injury is low. On the other hand, for the spine, the error ellipses of the different areas analyzed should be minimal and the ellipses have to be aligned, which would indicate that there are no torso flexions or lateral movements.
- The error ellipses of the shoulder markers allow for verifying the pendula movement of the back and the position adopted by the cyclists. According to the error ellipses obtained, the oval chainring improves the posture of the shoulders of the amateur cyclists studied. However, the results cannot be extrapolated to other cyclists, due to the low sample number analyzed.
- The error ellipses obtained from tracking the back markers allowed us to determine that the amateur cyclists tended to swing their backs when the required effort increased, unlike the professional cyclists who tended to improve their posture.
- It has been observed that the error ellipses obtained from the hip markers of the professional cyclists analyzed were similar, with collinear centers, and symmetric with respect to the vertical axis. This could indicate that the load distribution between their lower extremities is close to 50%.
- It was demonstrated that the uncertainty of the measurement device significantly affects the distribution of relative frequencies that meet the power required in the test. Therefore, it is recommended to consider the accuracy of the power meter to analyze the performance of the cyclists.
- The methodology used in the analysis of the power developed helped to visualize and compare the performance of the cyclists considering the uncertainty of the power meter, and penalized devices with low precision. In fact, low precision means greater uncertainty and higher power required, so the effort of the cyclist when using power meter devices with lower precision should be significantly higher.
- It was demonstrated that the maximum power alone is not a good parameter to measure the performance of the cyclist, since it is necessary to take into account the overall performance, such as the pedaling quality and the level of compliance with the completed power required.

## Figures and Tables

**Table 1:** Power analysis in the maximum effort stage of professional cyclist 1.

Properties	Oval Chainring	Circular Chainring
Mean power (W)	367	393
Relative standard deviation (%)	13	7.5
Maximum power (W)	438	427
Statistical mode (W)	322	388
Relative frequency of the mode (%)	6.6	5.9
Level of compliance with the completed power required (%)	51	93
